# Supplementary material for: Characteristics of sickle cell patients with frequent emergency department visits and hospitalizations
Source: PLoS One. 2021 Feb 22;16(2):e0247324. doi: 10.1371/journal.pone.0247324 (PMC7899345; doi:10.1371/journal.pone.0247324)
Supplement: S1 File — (DOCX) [file pone.0247324.s001.docx]

**Characteristics of Sickle Cell Patients with frequent ED Visits and Hospitalizations: Demographic, Clinical, Laboratory, and Psycho-social Aspects**

**Study ID:**

*Patient Information Questionnaire*

Please circle an answer for each question:

1. What is the highest degree or level of school you have completed?
   1. Some high school, no diploma
   2. High school graduate, diploma or equivalent (GED)
   3. Some college credit, no degree
   4. Trade, technical, or vocational training
   5. Associate’s or Bachelor’s degree
   6. Master’s, Doctorate, or Professional degree
2. Employment status - Are you currently:
   1. Employed for wages
   2. Out of work, looking for work
   3. Out of work, not currently looking for work
   4. Student
   5. Retired
3. What is your estimated annual household income?
   1. < $25,000
   2. $25,000 – $50,000
   3. $50,000 - $75,000
   4. $75,000 - $100,000
   5. >$100,000
4. What is your marital status?
   1. Single, never married
   2. Married or domestic partnership
   3. Widowed
   4. Divorced
   5. Separated
5. How many children do you have? (Please write in a number in the blank)

1. How many people live in your household? (Please write in a number in the blank)
