# Supplementary material for: Characteristics of sickle cell patients with frequent emergency department visits and hospitalizations
Source: PLoS One. 2021 Feb 22;16(2):e0247324. doi: 10.1371/journal.pone.0247324 (PMC7899345; doi:10.1371/journal.pone.0247324)
Supplement: Attachment — Submitted filename: Response to Reviewers.docx18-final.docx [file pone.0247324.s003.docx]

December 15, 2020

RE: PONE-D-20-26173

Monika R. Asnani, DM, PhD

Academic Editor

PLOS ONE

Dear Dr. Asnani:

We thank you and the reviewers for the very helpful comments and critiques on our manuscript titled “Characteristics of Sickle Cell Patients with Frequent Emergency Department Visits and Hospitalizations”, referenced above. We have incorporated the changes and corrections suggested by the reviewers, and are submitting the revised manuscript, with and without track changes, as recommended. Please find our responses to the Editors’ and reviewers’ points below:

**Editors’ coments:**

1. “Please provide additional details regarding participant consent. In the ethics statement in the Methods and online submission information, please ensure that you have specified both (1) whether consent was informed and (2) what type you obtained (for instance, written or verbal, and if verbal, how it was documented and witnessed)”

**This has been clarified in the manuscript, and a statement was added describing the written, informed consent process during clinic visits prior to enrollment.**

2. “Please include additional information regarding the survey *or questionnaire that was created by the authors and used in the study and ensure that you have provided sufficient details that others could replicate the analyses. For instance, if it is not under a copyright more restrictive than CC-BY, please include a copy, in both the original language and English, as Supporting Information”*

**The questionnaire that was administered to the patients to ascertain their socio-economic status is uploaded as supporting information.**

3. *“Please list the name and version of any software package used for statistical analysis, alongside any relevant references”*

**The data analyses were performed using R4.0.2 software. This has been added to the Statistical analysis section in the manuscript**

4. *“In your Results section, please include a table of relevant participant demographic details”*

**A table depicting the relevant demographic data for participants has been added (Table 1)**

5. *“Thank you for updating your data availability statement. You note that your data are available within the Supporting Information files, but no such files have been included with your submission. At this time we ask that you please upload your minimal data set as a Supporting Information file, or to a public repository such as Figshare or Dryad.
   Please also ensure that when you upload your file you include separate captions for your supplementary files at the end of your manuscript”.*

**An Excel file containing supplemental data will be uploaded along with this revision**

Cont..2/-

: 2 :

6. *Please ensure that you refer to Figure 2 in your text as, if accepted, production will need this reference to link the reader to the figure*”

**A reference to Figure 2 has been added to the manuscript**

7. *“We note your ethics statement states ethics approval was obtained from the Institutional Review Board of the Medical College of Georgia, while your manuscript states approval was obtained from Augusta University IRB.
   Before we can proceed, please update your ethics statement and methods and materials to reflect the correct IRB or Ethics Committee from whom approval was obtained”.*

**We apologize for this confusion. The institution underwent through several name changes in the past decade, and the Medical College of Georgia merged with another college and acquired the name Augusta University. The correct IRB is Augusta University IRB; this has been changed in the manuscript.**

**Reviewer 1:**

1. *“Please show a graph with plotted WBC, ANC, MCV and MCH values to corroborate the data in Table 3”*

**WBC, MCV, ANC have been plotted to corroborate the data in Table 3, and included as Figure 2**

2. “*Page 7, lane 161: “Two-sample t-tests were used to compared “ should be “Two-sample t-tests were used to	compare”*

**Page 7, line 161: “… t-tests were used to compared” has been corrected to “…compare”**

**Reviewer 2:**

1. *“The Title: I suggest a revision to " Characteristics of persons with sickle cell disease with frequent Emergency Department ....The phrase "Sickle Cell Patients " does not give an indication that a variety of genotypes were studied and should not be used.
   see also line 200 - patients with sickle cell disease.”*

**We kept the term “Characteristics of Sickle Cell Patients…” in the title, because we used the term “sickle cell patients” broadly to encompass all genotypes (SS, SC etc.) as indicated in the Table showing the demographic features**

2. *“Reference#1: I would suggest that the primary reference, Steiner et al be used. The data in the reference ( Singh et al could be used separately to support the text”*

**Steiner et al has been used as reference** **1**

3. *The aims could be stated more specifically: For example what differences were being examined were they genotype, hydroxyurea use, previous opioid use, haematological and biochemical variables etc.*

**A sentence has been added to clearly specify the aims, on page 3, lines 72-73**

4. *Typographical error - line 73 - "Hopes" please change to "Hope";*

**Typographical error ‘hopes” has been corrected to “hope”, line 74**

5. *Methods: line 87 - change to "within the last calendar year at the time of recruitment"*

**Line 87, changed to “…within the last calendar year at the time of recruitment”**

Cont..3/-

: 3 :

6. *Line89 - change to within the last 3 months prior to recruitment.*

**Line 91 “…within the last 3 months prior to recruitment” added**

7. *Line 110: Change to " Blood Samples for Haematological or biochemical analysis was not taken as a part of the study" or a similar phrase*

*Please indicate the period of time that results were accepted retrospectively from the time of enrollment. Please state whether patients were well at the time these results were documented and if the results obtained were considered to be "steady state results"*

**Line 115-117: “Blood samples for ……. as part of the study” added, and the period of time that results were accepted retrospectively has been clarified as the time of previous clinic visit, which is usually 2 months prior, and also it was confirmed that the subjects were at “steady state” at that time**

8. *The examiners conducting quantitative sensory testing were well experienced and the results were generated from only 2 examiners however please state whether tests were done for inter-observer reliability.*

**A statement has been added to specify training of the examiners for quantitative sensory testing to address inter-observer reliability (Lines 137-138)**

9. *Results: Please state the average duration of hydroxyurea therapy in patients on hydroxyurea and also if they were at maximum tolerated dose.*

**Duration of HU therapy was not specified for the subjects enrolled; however, those who were prescribed HU were “on HU” for >1 year**

10. *line 200-202 should be placed in the discussion*

**Lines 200-202, 252-256 and 265-267 have been moved to discussion.** Thanks for the suggestion!

11. *The legend submitted for figure 1 can be more concise. Please consider making this shorter.*

**Legend for Figure 1 has been revised and shortened**

12. *Was a multivariate analysis of the main outcome considered?*

**Multivariate analysis of the main outcome was not considered due to limited sample size**

13. *Was the use of hydroxyurea between groups statistically significant? Please state this.*

**HU use was 76% in the frequent utilizer group, and 77.8% in the non-frequent utilizer group; the difference was not statistically significant (p=0.6367 with Chi-squared test)**

14. *Discussion: The findings regarding hydroxyurea should be made cautiously. We are not told how long patients were on HU or if they were at maximum tolerated dose to assess parameters such as MCV.
    The observations regarding hydroxyurea use even though they may be interesting should be stated a bit more cautiously as details concerning use was not documented in the paper.
    Generally, the discussion needs to be more concise and directed on findings from the study.*

**HU use is based upon prescription of the medication, and does not take into account issues related to adherence. The discussion with regards to HU has been modified along the lines suggested by the reviewer**

Cont..4/-

: 4 :

**Reviewer 3:**

1. *Introduction: Line 58- word missing. Should be “carried out in”*

**Line 58: “..carried out in” added**

2. *Figure:
   - It should be edited to improve contrast and crispness of the text.
   - The statistically significant finding should be indicated on the figure.*

**The figure has been revised to improve the contrast and significant findings have been indicated**

3. *Sample size
   Researchers have not given any reasons for including 25 high utilization and 9 low utilization subjects; on what basis was number of cases and controls decided? Was this an audit of all eligible patients? How many refused? Was there a primary outcome used to calculate a required sample size? What determined the length of time for subject accrual?*

**The sample size was not based on a pre-calculated power analysis. The number of high utilizers and low utilizers were selected from patients who receive their care at our institution. Frequent utilizers were defined as those with >6 ED visits/hospitalizations in a year, based upon CSSCD data, as mentioned in the introduction, and 25 such patients were identified.**

4. *Methods:
   What was the range of the period of time between blood investigations and the study visit?*

**For the range of time between blood draw and study visit, see item #7 in response to Reviewer 2’s comments above**

5. *Did the subjects access all their care at the study sites? Could they have used prescription opioids not accounted for in the chart review?*

**It is highly unlikely that the subjects received opioid prescriptions from other providers/institutions, as they were closely monitored in the Georgia Prescription Monitoring System**

6. *Were there any other methods which could have been used to assess sensation? Electronic von Frey has been reported to be more reliable and rapid than VFM in exploring mechanical pain thresholds”*

**We did not use electronic Von Frey assessment.**

7. *Statistical methods:
   - Was any adjustment made for multiple comparisons?*

**We did not perform adjustment for multiple comparisons due to limited sample size**

8. *- T tests were done. Were the distributions of the normal variables normal?*

- It would be helpful if the categorization of variables was provided. For example, how was education categorized?

**We checked for normality with Kolmogorov-Smirnov (K-S) test before the t-tests and K-S test did not reject the normality assumption. Education was “caterorized as college or associate degree” and “no college or associate degree”**

Cont..5/-

**: 5 :**

9. *- Was the absence of multivariate analysis a function of the sample size?*

**The absence of multivariate analysis was a result of the sample size.**

10. *Results:
    Some aspects of the results are interpretations of the data and may be better placed in the discussion. For example “The combination of lower pain thresholds and considerably higher opioid use in the high utilizer group suggests that opioid induced hyperalgesia may be contributing to increased pain sensitivity in these patients. Increased baseline sensitivity to pain could predispose SCD patients to developing more frequent acute painful crises, leading to the increased number of ED visits and hospitalizations seen in patients from the high utilizer group.”*

**Some sections in the results have been moved to discussion, where they are more appropriate. See also item #10 in response to Reviewer 2.**

11. *Discussion:
    On what basis was an assessment made of the direction of relationships? Cross-sectional studies demonstrate associations but the direction of relationships is usually elucidated by longitudinal assessment.*

**Discussion has been modified (Lines 424-429) to include the possible role of genetic and environmental factors contributing to disease severity and high utilization, as suggested by the reviewer.**

12. *The authors should discuss the reasons why only the ulnar measurement showed statistically significant differences. Could this have been related to sample size?*

**The observation that only the ulnar measurement in QST showed significant difference between two groups could be a reflection of limited sample size.**

We hope that the revised manuscript will be acceptable for publication, and look forward to hearing from you. Please let me know if there are further questions.

Sincerely,

Abdullah Kutlar MD Professor of Medicine

Dr. William N. Agostas and Josephine R. Agostas Chair in Internal Medicine Division of Hematology/Oncology& Stem Cell Transplantation

Director, Center for Blood Disorders

Medical College of Georgia, Augusta University 989 St. Sebastian Way, EF 145C

Augusta, GA 30912

Phone: (706) 721-2171|Fax: (706) 721-7226

[akutlar@augusta.edu](mailto:akutlar@augusta.edu)
