# Supplementary material for: Characteristics of sickle cell patients with frequent emergency department visits and hospitalizations
Source: PLoS One. 2021 Feb 22;16(2):e0247324. doi: 10.1371/journal.pone.0247324 (PMC7899345; doi:10.1371/journal.pone.0247324)
Supplement: Attachment — Submitted filename: ResubmissionCommentsAandE110121.docx [file pone.0247324.s004.docx]

Plos one

Reviewer 2

8. The sentence added does not obviate the benefit of inter observer reliability testing. This is a limitation which the researchers should acknowledge.
