# Supplementary material for: Characteristics of sickle cell patients with frequent emergency department visits and hospitalizations
Source: PLoS One. 2021 Feb 22;16(2):e0247324. doi: 10.1371/journal.pone.0247324 (PMC7899345; doi:10.1371/journal.pone.0247324)
Supplement: Attachment — Submitted filename: Response to Reviewers2.docx [file pone.0247324.s005.docx]

February 2, 2021

RE: PONE-D-20-26173R1

Monika R. Asnani, DM, PhD

Academic Editor

PLOS ONE

Dear Dr. Asnani:

We thank you and the reviewers for the very helpful comments and critiques on our manuscript titled “Characteristics of Sickle Cell Patients with Frequent Emergency Department Visits and Hospitalizations”, referenced above. We are delighted that the reviewers have commented favorably on the revised manuscript. We have incorporated the final changes and corrections suggested by the reviewers, and are submitting the revised manuscript, with and without track changes, as recommended. Please find our responses to the Editors’ and reviewers’ points below:

**Reviewer 2:**

*The manuscript seeks to determine factors which contribute to high health care utilization in sickle cell disease which is a very important topic. The author's responses are satisfactory and the revisions adequate, however please see two minor comments:
1. Please correct typos in lines 42, 228 and 324.*

**Typos in lines 42, 228and 324 have been corrected. We thank the reviewer for noting this.**

2. *Please clarify labs further in manuscript as follows: ....from the patient’s prior clinic visit ....Please add "which was usually 2 months before enrollment". The latter comment was in the response to the reviewer and is duly noted but should also be placed in the manuscript*.

**The phrase “…which was usually 2 months before enrollment” was added in line 112**

**Reviewer 3:**

*The sentence added does not obviate the benefit of inter observer reliability testing. This is a limitation which the researchers should acknowledge.*

**A sentence acknowledging the possibility of interobserver variability has been added to the discussion.**

We hope that the revised manuscript will be acceptable for publication, and look forward to hearing from you. Please let me know if there are further questions.

Sincerely,

Abdullah Kutlar MD Professor of Medicine

Dr. William N. Agostas and Josephine R. Agostas Chair in Internal Medicine Division of Hematology/Oncology& Stem Cell Transplantation

Director, Center for Blood Disorders

Medical College of Georgia, Augusta University 989 St. Sebastian Way, EF 145C

Augusta, GA 30912

Phone: (706) 721-2171|Fax: (706) 721-7226

[akutlar@augusta.edu](mailto:akutlar@augusta.edu)
